# Recent Advances in Kidney Disease Diagnosis and Treatment: Bridging Molecular Insights and Clinical Practice

**DOI:** 10.3390/diagnostics16040549

**Published:** 2026-02-13

**Authors:** Kenji Tsuji

**Affiliations:** Department of Nephrology, Rheumatology, Endocrinology and Metabolism, Okayama University Graduate School of Medicine, Dentistry and Pharmaceutical Sciences, 2-5-1 Shikata-cho, Okayama 700-8558, Japan; ktsuji@s.okayama-u.ac.jp; Tel.: +81-86-235-7235

Chronic kidney disease (CKD) is a major global public health problem, affecting hundreds of millions of patients worldwide and imposing substantial economic costs [1]. Because of the asymptomatic early stages and heterogeneous clinical pathophysiology, CKD remains underdiagnosed until advanced stages, at which point therapeutic options are limited, resulting in poor patient outcomes. Consequently, the development of sensitive diagnostic tools for early CKD detection, reliable biomarkers of disease progression, and effective therapeutic strategies remains a central challenge in nephrology. Since the mid-2010s, substantial progress has been achieved in the diagnosis and treatment of kidney diseases. Advances in molecular diagnostics, metabolomics, and genetic profiling have deepened our understanding of the mechanisms of kidney disease. In parallel, the therapeutic strategy for CKD has expanded from conventional conservative management, such as blood pressure control and dietary therapy, to include disease-modifying strategies that directly target inflammation and fibrosis, which represent the common final pathways in CKD progression. Following the establishment of the “four pillars” of Diabetic Kidney disease (DKD) management, renin-angiotensin system blockade, SGLT2 inhibitors, non-steroidal mineralocorticoid receptor antagonists, and glucagon-like peptide-1 receptor agonists, increasing attention has been directed toward adjunctive therapies aimed at managing residual risk [2]. Chronic inflammation, oxidative stress, immune cell infiltration, and the activation of profibrotic signaling pathways are now recognized as central common pathways of CKD progression across etiologies [3]. Accordingly, multiple pharmacological approaches targeting these pathways have entered clinical development [4], including endothelin receptor antagonists (ERAs) [5], soluble guanylate cyclase stimulator [6], Janus kinase (JAK)-STAT pathway inhibitors [7], aldosterone synthase inhibitors [8], the NOD-like receptor family, pyrin-domain-containing 3 (NLRP3) inflammasome inhibitors, C-C chemokine receptor type 2 and 5 (CCR2/CCR5) antagonists [9], apoptosis-signal-regulating kinase 1 (ASK1) inhibitors [10], nuclear factor erythroid 2-related factor 2 (Nrf2) activators [11], and agents modulating microRNA-mediated fibrotic responses [12].

ERAs have produced beneficial effects, including reductions in proteinuria and the attenuation of inflammatory and fibrotic responses, particularly when combined with SGLT2 inhibitors [5,13]. Endothelin-1 (ET-1) is a 21-amino-acid peptide that was first identified in 1988 and is a potent vasoconstrictor in humans. The kidney is one of the major organs responsible for ET-1 production, and ET-1 is secreted by various kidney cell types, including glomerular endothelial cells, tubular epithelial cells, and interstitial fibroblasts. ET-1 exerts biological effects primarily through two G-protein-coupled receptors, endothelin type A (ETA) and endothelin type B (ETB) receptors [14,15]. ETA receptors are predominantly expressed in vascular smooth muscle cells and fibroblasts, where their activation induces vasoconstriction, cellular proliferation, inflammation, and fibrogenesis. In contrast, ETB receptors are mainly located on endothelial cells and mediate vasodilation and natriuresis through the production of nitric oxide and prostacyclin. In patients with CKD, ET-1 expression is markedly upregulated, leading to the activation of ETA signaling, which induces renal vasoconstriction, reduces renal blood flow, and exacerbates intrarenal hypoxia [16]. In addition, ET-1 contributes to the development of proteinuria by disrupting the endothelial glycocalyx in glomerular capillaries [17]. ET-1 also promotes the recruitment of macrophages and T cells as well as enhances the production of pro-inflammatory cytokines such as tumor necrosis factor-α (TNF-α) and interleukin-6 (IL-6) [18]. These processes collectively drive fibroblast activation and myofibroblast differentiation, resulting in tubulointerstitial fibrosis and CKD progression. ERAs exert multifaceted reno-protective effects by blocking ET-1 signaling, leading to reductions in proteinuria, the stabilization of renal hemodynamics, and the suppression of inflammation and fibrosis. Although not all candidate agents have produced definitive reno-protective benefits in late-phase trials, these efforts collectively reflect a paradigm shift toward mechanism-based and combination therapies that aim to slow CKD progression. These advances highlight the growing incorporation of molecular pathophysiology into clinical trials and routine practice, enabling the selection of more precise and individualized therapeutic strategies in nephrology. In addition, improvements in renal replacement therapies, including hemodialysis, peritoneal dialysis, renal transplantation, and regenerative approaches, have contributed to increased patient survival and quality of life. Despite these advances, important gaps remain between mechanistic insights and their translation into routine clinical practice.

This second edition of the Special Issue “*Recent Advances in Diagnosis and Treatment of Kidney Diseases*” was designed to highlight contemporary progress in kidney disease diagnostics and therapeutics, with a particular focus on CKD, dialysis-related complications, and renal transplantation, and addressed critical aspects of kidney disease from molecular mechanisms to clinical outcomes. Several contributions in this Special Issue focus on patients undergoing hemodialysis, a population characterized by complex metabolic, endocrine, and cardiovascular disturbances. One original study explored the drivers of hypothyroidism in patients on hemodialysis, highlighting a frequently overlooked but clinically relevant complication [19]. Thyroid dysfunction in patients on dialysis is associated with increased cardiovascular risk and mortality; however, the underlying mechanisms remain incompletely understood. By identifying the contributing clinical and biochemical factors, this study provides valuable insights that may inform improved screening and management strategies in this vulnerable population. Another original investigation explored the association between serum phenylacetylglutamine levels and aortic stiffness in patients on hemodialysis [20]. Cardiovascular disease remains the leading cause of death in patients with CKD on dialysis, and arterial stiffness is a key predictor of adverse cardiovascular outcomes. The identification of phenylacetylglutamine, a gut microbiota-derived uremic metabolite, as a potential risk factor highlights the growing importance of metabolic and microbiome-related pathways in CKD-associated cardiovascular complications. Despite substantial progress, several diagnostic and therapeutic challenges persist in kidney disease care. Traditional biomarkers such as serum creatinine and estimated glomerular filtration rate (eGFR) lack sensitivity, especially for detecting early disease detection and capturing the complexity of CKD pathophysiology. Moreover, many CKD complications, including metabolic acidosis, cardiovascular disease, and endocrine disturbances, are multifactorial, highlighting the need for more integrative diagnostic approaches. The narrative reviews included in this Special Issue directly address these gaps. One review focuses on emerging biomarkers and advanced diagnostic strategies in CKD, emphasizing the role of multiomics technologies and artificial intelligence [21]. By integrating genomics, transcriptomics, proteomics, metabolomics, and advanced imaging with machine-learning-based analytics, these approaches offer promising strategies for earlier disease detection, higher-accuracy risk stratification, and personalized therapeutic decision-making. This review also critically discusses the current limitations, including data integration, standardization, and clinical validation, that must be addressed before widespread implementation. Collectively, the articles in this Special Issue illustrate how advances in molecular diagnostics, metabolism, pharmacogenomics, and data-driven approaches are redefining the current approaches in nephrology. Importantly, the articles advance our understanding of disease mechanisms, which can be translated into clinically meaningful strategies for risk assessment, treatment optimization, and complication management.

By covering diverse aspects of kidney disease, ranging from dialysis-related metabolic and cardiovascular complications to transplant immunology and advanced diagnostics, this Special Issue underscores the need for a multidisciplinary approach to kidney disease. Future research on kidney disease diagnostics and treatment should prioritize longitudinal and real-world studies that capture disease trajectories over time. Integrating molecular biomarkers with clinical phenotypes will be essential for advancing precision nephrology. In addition, the development of AI-assisted diagnostic tools and genotype-guided therapies would be promising for improving individualized patient care. Finally, close collaboration among basic scientists, clinicians, and data scientists will be critical to overcoming the existing translational hurdles. In conclusion, this Special Issue provides a timely overview of the recent advances in the diagnosis and treatment of kidney diseases while highlighting persistent challenges and future opportunities. We hope that these studies will stimulate further research and contribute to the continued evolution of diagnostic and therapeutic strategies in nephrology from concept to routine clinical care.

## References

[1] Francis A., Harhay M.N., Ong A.C.M., Tummalapalli S.L., Ortiz A., Fogo A.B., Fliser D., Roy-Chaudhury P., Fontana M., Nangaku M. (2024). Chronic kidney disease and the global public health agenda: An international consensus. Nat. Rev. Nephrol..

[2] Han S., Kim S. (2024). A New Era in Diabetic Kidney Disease Treatment: The Four Pillars and Strategies to Build Beyond. Electrolyte Blood Press.

[3] Stenvinkel P., Chertow G.M., Devarajan P., Levin A., Andreoli S.P., Bangalore S., Warady B.A. (2021). Chronic Inflammation in Chronic Kidney Disease Progression: Role of Nrf2. Kidney Int. Rep..

[4] Yi T.W., Sridhar V.S., Scott J., Nardone M., Cherney D. (2026). Next-generation therapeutics for diabetic kidney disease. Nat. Rev. Nephrol..

[5] Heerspink H.J.L., Kiyosue A., Wheeler D.C., Lin M., Wijkmark E., Carlson G., Mercier A.K., Astrand M., Ueckert S., Greasley P.J. (2023). Zibotentan in combination with dapagliflozin compared with dapagliflozin in patients with chronic kidney disease (ZENITH-CKD): A multicentre, randomised, active-controlled, phase 2b, clinical trial. Lancet.

[6] Xu Y., Wu X., Ge M., Liang K., Xing J., Zhang P., Li H., Guo Z., Mei X. (2025). Soluble guanylate cyclase modulators-a novel therapeutic approach for diabetic kidney disease. Ren. Fail..

[7] Tuttle K.R., Brosius F.C., Adler S.G., Kretzler M., Mehta R.L., Tumlin J.A., Tanaka Y., Haneda M., Liu J., Silk M.E. (2018). JAK1/JAK2 inhibition by baricitinib in diabetic kidney disease: Results from a Phase 2 randomized controlled clinical trial. Nephrol. Dial. Transpl..

[8] Merlo M., Zoccatelli F., Costa G., Panepinto L., Friso S., Marzano L. (2026). Aldosterone synthase inhibitors across the translational spectrum: Mechanistic foundations and emerging clinical applications. J. Intern. Med..

[9] Lefebvre E., Moyle G., Reshef R., Richman L.P., Thompson M., Hong F., Chou H.L., Hashiguchi T., Plato C., Poulin D. (2016). Antifibrotic Effects of the Dual CCR2/CCR5 Antagonist Cenicriviroc in Animal Models of Liver and Kidney Fibrosis. PLoS ONE.

[10] Heerspink H.J.L., Perkovic V., Tuttle K.R., Pergola P.E., Mahaffey K.W., Patel U.D., Ishida J.H., Kuo A., Chen F., Kustra R. (2024). Selonsertib in Patients with Diabetic Kidney Disease: A Phase 2b Randomized Active Run-In Clinical Trial. J. Am. Soc. Nephrol..

[11] Nangaku M., Kanda H., Takama H., Ichikawa T., Hase H., Akizawa T. (2020). Randomized Clinical Trial on the Effect of Bardoxolone Methyl on GFR in Diabetic Kidney Disease Patients (TSUBAKI Study). Kidney Int. Rep..

[12] Gale D.P., Gross O., Wang F., Esteban de la Rosa R.J., Hall M., Sayer J.A., Appel G., Hariri A., Liu S., Maski M. (2024). A Randomized Controlled Clinical Trial Testing Effects of Lademirsen on Kidney Function Decline in Adults with Alport Syndrome. Clin. J. Am. Soc. Nephrol..

[13] Anguiano L., Riera M., Pascual J., Soler M.J. (2015). Endothelin Blockade in Diabetic Kidney Disease. J. Clin. Med..

[14] Liang F., Li G., Chen K., Chen J., He J., He Y. (2025). Endothelial Dysfunction and Therapeutic Advances in Chronic Kidney Disease. Diabetes Metab. Res. Rev..

[15] Ma X., Liang Y., Chen W., Zheng L., Lin H., Zhou T. (2025). The role of endothelin receptor antagonists in kidney disease. Ren. Fail..

[16] Afsar B., Afsar R.E., Dagel T., Kaya E., Erus S., Ortiz A., Covic A., Kanbay M. (2018). Capillary rarefaction from the kidney point of view. Clin. Kidney J..

[17] Ebefors K., Wiener R.J., Yu L., Azeloglu E.U., Yi Z., Jia F., Zhang W., Baron M.H., He J.C., Haraldsson B. (2019). Endothelin receptor-A mediates degradation of the glomerular endothelial surface layer via pathologic crosstalk between activated podocytes and glomerular endothelial cells. Kidney Int..

[18] Sasser J.M., Sullivan J.C., Hobbs J.L., Yamamoto T., Pollock D.M., Carmines P.K., Pollock J.S. (2007). Endothelin A receptor blockade reduces diabetic renal injury via an anti-inflammatory mechanism. J. Am. Soc. Nephrol..

[19] Ratiu I.A., Babeș E.E., Georgescu L.M., Hocopan O., Dejeu D., Moisa C., Gavra D.N., Ratiu C.A. (2026). Understanding the Drivers of Hypothyroidism in Patients Undergoing Chronic Hemodialysis. Diagnostics.

[20] Su I.M., Wu T.J., Liu C.H., Lin Y.L., Hsu B.G. (2025). Serum Phenylacetylglutamine Is a Potential Risk Factor for Aortic Stiffness in Patients with Chronic Hemodialysis. Diagnostics.

[21] Alobaidi S. (2025). Emerging Biomarkers Advanced Diagnostics in Chronic Kidney Disease: Early Detection Through Multi-Omics, A.I. Diagnostics.

